# The heat shock factor GhHSFA4a positively regulates cotton resistance to *Verticillium dahliae*

**DOI:** 10.3389/fpls.2022.1050216

**Published:** 2022-11-03

**Authors:** Lu Liu, Di Wang, Chao Zhang, Haiyang Liu, Huiming Guo, Hongmei Cheng, Enliang Liu, Xiaofeng Su

**Affiliations:** ^1^ Biotechnology Research Institute, Chinese Academy of Agricultural Sciences, Beijing, China; ^2^ Center for Advanced Measurement Science, National Institute of Metrology, Beijing, China; ^3^ State Key Laboratory of North China Crop Improvement and Regulation, College of Life Science, Hebei Agricultural University, Baoding, China; ^4^ Institute of Plant Protection, Xinjiang Academy of Agricultural Sciences, Urumqi, China; ^5^ Hainan Yazhou Bay Seed Lab, Sanya, China; ^6^ Institute of Grain Crops, Xinjiang Academy of Agricultural ScienceS, Urumqi, China

**Keywords:** cotton, *Verticillium* wilt, *Verticillium dahliae*, heat shock factors, GhHSFA4a

## Abstract

Heat shock factors (HSFs) play a crucial role in the environmental stress responses of numerous plant species, including defense responses to pathogens; however, their role in cotton resistance to *Verticillium dahliae* remains unclear. We have previously identified several differentially expressed genes (DEGs) in *Arabidopsis thaliana* after inoculation with *V. dahliae*. Here, we discovered that *GhHSFA4a* in *Gossypium hirsutum* (cotton) after inoculation with *V. dahliae* shares a high identity with a DEG in *A. thaliana* in response to *V. dahliae* infection. Quantitative real-time PCR (qRT-PCR) analysis indicated that *GhHSFA4a* expression was rapidly induced by *V. dahliae* and ubiquitous in cotton roots, stems, and leaves. In a localization analysis using transient expression, GhHSFA4a was shown to be localized to the nucleus. Virus-induced gene silencing (VIGS) revealed that downregulation of *GhHSFA4a* significantly increased cotton susceptibility to *V. dahliae.* To investigate *GhHSFA4a-*mediated defense, 814 DEGs were identified between *GhHSFA4a-*silenced plants and controls using comparative RNA-seq analysis. The Kyoto Encyclopedia of Genes and Genomes (KEGG) analysis showed that DEGs were enriched in “flavonoid biosynthesis”, “sesquiterpenoid and triterpenoid biosynthesis”, “linoleic acid metabolism” and “alpha-linolenic acid metabolism”. The expression levels of marker genes for these four pathways were triggered after inoculation with *V. dahliae*. Moreover, *GhHSFA4a*-overexpressing lines of *A. thaliana* displayed enhanced resistance against *V. dahliae* compared to that of the wild type. These results indicate that *GhHSFA4a* is involved in the synthesis of secondary metabolites and signal transduction, which are indispensable for innate immunity against *V. dahliae* in cotton.

## Introduction

As a major source of fiber and oil, cotton forms the economic backbone of several developing countries (Wu et al., 2022). Cotton yields are reduced by numerous diseases, the most destructive being cotton *Verticillium* wilt (CVW), sometimes called the “cancer” of cotton (Shaban et al., 2018). Approximately 40% of the cotton-growing areas in China are affected by this disease, causing annual losses of 250 to 310 million US dollars (Wang et al., 2016; Gong et al., 2017). The fungus *Verticillium dahliae*, the cause of CVW, has numerous races and produces microsclerotia that are highly resistant to biotic as well as abiotic stresses and can survive in the soil for decades (Deketelaere et al., 2017; Harting et al., 2020). In addition, chemical and biotechnological methods are ineffective in controlling pathogens and reducing losses (Zhang et al., 2018). Therefore, identifying resistance genes and breeding resistant varieties of cotton with broad-spectrum resistance are primary control measures (Zhang et al., 2014).

During the activation of plant defense responses in resistant plants, transcription factors (TFs) play an essential role in modulating downstream gene expression to induce stress adaptation signaling or strategic regulators in pathways (Udvardi et al., 2007; Kilian et al., 2012). The R2R3-type MYB protein GhMYB36 is involved in *Verticillium* wilt resistance in *Arabidopsis* and cotton by positively regulating the expression of PR1 (Liu et al., 2022). Two TFs, vascular plant one-zinc finger 1 (OsVOZ1) and OsVOZ2, are involved in rice resistance to *Magnaporthe oryzae* by regulating the accumulation of nucleotide-binding leucine-rich repeat (NLR) protein Piz-t (Andrási et al., 2021).

TFs, including heat shock factors (HSFs), ERF/AP2, bZIP, MYB, MYC, NAC, and WRKY are classified into different families based on their functional regions (Alves et al., 2014; Guo et al., 2016): DNA-binding regions, transcriptional regulatory regions (including activation and repression regions), oligomerization sites, and localization signals (Franco-Zorrilla and Solano, 2017). HSFs have a DNA-binding domain (DBD) and oligomerization domain (OD). Usually, the DBD is a highly conserved segment and the OD consists of two hydrophobic heptapeptide repeat regions, HR-A/B. HSFs can be classified into three categories, A, B, and C, based on the difference between DBD and HR-A/B. HSFs specifically bind to heat shock elements (HSEs), which are highly conserved promoter regions in heat shock protein (*HSP*) genes, and bind to other TFs to form a transcriptional complex that regulates the expression of *HSP* genes (von Koskull-Döring et al., 2007; Scharf et al., 2012). Previous studies on HSF function have mainly focused on plant resistance to abiotic stresses, such as drought, cold, heat, salt, and oxidative stress. ZmHSF08 has been shown to negatively regulate several stress/ABA-responsive genes under salt and drought stress conditions in maize (Wang et al., 2021). The expression of *TaHSFA2e-5D* is highly upregulated in wheat seedlings by heat, cold, drought, high salinity, and multiple phytohormones (Bi et al., 2022).

Among the results of several studies investigating the HSF function in plant disease resistance, the transcription factor CIGR2 suppresses excessive cell death caused by rice blast fungus by activating the expression of *OsHSF23* in rice (Tanabe et al., 2016). MeHSF3 directly targets the HSEs of *enhanced disease susceptibility 1* (*EDS1*) and *pathogenesis-related gene 4* (*PR4*), which positively regulate the immune response of *Manihot esculenta* against *Xanthomonas axonopodis pv. manihotis* (*Xam*) (Wei et al., 2018). After infection of tomato roots with *Meloidogyne incognita*, HSFA1a increases the expression of the respiratory burst oxidase homolog (RBOH) family protein Wfi1, initiating the HSFA1a-Wif1 cascade reaction and triggering a reactive oxygen species (ROS) burst (Zhou et al., 2018). The resistance response of pepper against *Pseudomonas solanacearum* involves CaHSFB2a through a positive feedback loop involving CaWRKY6 and CaWRKY40 (Ashraf et al., 2018). Overexpression OsHSFB4d in rice exhibited enhanced resistance to *Xanthomonas oryzae* pv. *oryzicola* as well as *Xanthomonas oryzae* pv. *oryzae* and an increased expression of *OsHsp18.0-CI* as well as pathogenesis-related genes (Yang et al., 2020).

Although multiple lines of evidence support a critical regulatory function of HSFs in plant disease resistance pathways, the role of HSFs in cotton resistance to *V. dahliae* remains unknown. In our previous study, we identified a number of differentially expressed genes (DEGs) through in-depth transcriptomic and metabolic analyses of *A. thaliana* infected with *V. dahliae* (Su et al., 2018) and used BlastP to identify the homolog of a DEG in *Gossypium hirsutum*, the heat shock factor A-4a-like gene (*GhHSFA4a*, XM_016875437.1). In the present study, we investigated the function of *GhHSFA4a* and analyzed (1) the physicochemical properties as well as phylogeny of GhHSFA4a, (2) the expression pattern of *GhHSFA4a* after infection with *V. dahliae*, (3) the subcellular location of GhHSFA4a in tobacco cells, (4) the disease resistance phenotype in cotton seedlings after virus-induced silencing (VIGS) of *GhHSFA4a*, (5) determined the defense pathway mediated by GhHSFA4a based on transcriptome and quantitative real-time PCR (qRT-PCR) analyses, and (6) generated *GhHSFA4a*-overexpressing *A. thaliana* plants as well as evaluated their resistance to *V. dahliae.*

## Materials and methods

### Plant materials, fungal strains, and growth conditions

Seedlings of cotton cultivar Coker 312 were grown in an autoclaved mixture of nutrient soil and vermiculite (1:1) in a greenhouse at 25 ± 2°C, 75 ± 5% relative humidity, with a photoperiod 16 h light/8 h dark. Seedlings of *Arabidopsis* (Columbia-0) were grown in nutrient-supplemented soil with 16 h light/8 h dark at 22 °C and 60% relative humidity. Strain V991 of *V. dahliae*, a highly virulent and defoliating pathogenic strain provided by Prof. Xiaofeng Dai of the Institute of Plant Protection, Chinese Academy of Agricultural Sciences (CAAS), was cultured on potato dextrose agar (PDA) at 25°C for 7 - 10 days or in liquid complete medium (CM) at 200 rpm and 25°C, for 2 - 3 days to be used as inoculum.

### Bioinformatic analysis

The amino acid sequences of GhHSFA4a and homologous proteins from *A. thaliana*, *Solanum lycopersicum*, *Malus domestica*, *G. hirsutum*, *Fragaria vesca*, *Oryza sativa*, and *Zea mays* were downloaded from the NCBI database (https://www.ncbi.nlm.nih.gov/). A phylogenetic tree was constructed using the neighbor-joining method in the MEGA software (version 5.0, Auckland, New Zealand). Amino acid sequence alignment and mapping were performed using ClustalW and ESPript 3.0 (https://espript.ibcp.fr/ESPript/ESPript/). The PlantCARE database was used to analyze *cis*-acting elements in a 2000-bp region located upstream of *GhHSFA4a* (Lescot et al., 2002), and the motif was drawn using TBtools (https://github.com/CJ-Chen/TBtools-Manual). Protein structure was predicted and analyzed using the AlphaFold Protein Structure Database (https://alphafold.com/) and UCSF Chimera (Pettersen et al., 2004), while subcellular locations were predicted using Uniport (https://www.uniprot.org).

### Subcellular localization and confocal microscopy

In combination with the predicted subcellular localization results from the online platform WoLFPROST (https://wolfpsort.hgc.jp/), the full-length coding sequence (CDS) of the *GhHSFA4a* gene was cloned using 1132-GhHSFA4a-F/R (**Table S1**) and inserted into the expression vector *pYBA1132* (BamHI and EcoRI) containing the green fluorescent protein gene (*GFP*). The construct was inserted into *Agrobacterium tumefaciens* EHA105, which was subsequently used for agrobacterial transformation of *Nicotiana benthamiana*. Cells were observed under an LSM980 confocal laser-scanning microscope (Zeiss, Jena, Germany). The nuclear marker *H2B-mCherry* was provided by Prof. Lei Wang from the Biotechnology Research Institute, CAAS (Lu et al., 2022).

### Pathogenicity assay

When cotton seedlings had two true leaves, plants were carefully uprooted and the roots were submerged in a suspension of *V. dahliae* conidia (10^7^ cfu/mL) for 5 min. Each group consisted of five plants. Plants were replanted and grown for 15 days in the greenhouse, and their disease severity was scored on a 0 to 4 scale: 0, no disease; 1, both cotyledons yellow and true leaves were disease-free; 2, both cotyledons had symptoms and 1-3 true leaves had symptoms; 3, more than 5 leaves, including cotyledons, had symptoms; level 4, all leaves had symptoms, leaves had fallen off, and the plant was dead. The disease index (DI) was calculated as [∑ (number of diseased plants at each severity × disease score)/(total checked plants × 4)] × 100 (Li et al., 2020).

### VIGS in cotton and RNA-seq analysis

The pTRV2 vector was digested using restriction endonucleases EcoRI and BamHI. The VIGS interference fragment in *GhHSFA4a* was amplified using the primers pTRV2-GhHSFA4a-F/R (**Table S1**) and then ligated using the seamless cloning method. The pTRV2::*GhHSFA4a* plasmid was inserted into *A. tumefaciens* GV3101 for the VIGS assay (Gao et al., 2011). Following injection, when the second true leaf on the pTRV2::*CLA1* positive control plants developed photobleaching, silencing efficiency was assessed using qRT-PCR with primers qGhHSFA4a-F/R (**Table S1**). The pTRV2::*00* and pTRV2::*GhHSFA4a* cotton were sampled, subjected to RNA-seq, and analyzed (Sajjad et al., 2021). Cotton *polyubiquitin* (*UBQ*, LOC107925174) was used as the housekeeping gene (**Table S1**).

To assess the integrity and total amount of RNA, an Agilent 2100 Bioanalyzer (Santa Clara, CA, USA) and Qubit 2.0 (Life Technologies, Carlsbad, CA, USA) were used to assess the quality of total RNA. The mRNA was enriched using oligo (dT) magnetic beads and randomly interrupted using NEB Fragmentation Buffer, and each sample was pooled in three replicates to form a single RNA sequencing library and the NEBNext^®^ Ultra™ RNA Library Prep Kit for Illumina^®^ Kit (Illumina, San Diego, CA, USA). Transcriptome sequencing was performed using an Illumina NovaSeq 6000 system. Clean reads for subsequent analyses were obtained after raw data filtering, sequencing error rate examination, and GC content distribution determination. The position information of the gene alignment on *G. hirsutum* TM-1 was analyzed to obtain the original read counts. Using DESeq, we obtained normalized read counts, which were screened for DEGs using the | log2 (Fold change) | ≥ 1 and padj ≤ 0.05 standard (Purayil et al., 2020). Gene Ontology (GO), Kyoto Encyclopedia of Genes and Genomes (KEGG), and Gene Set Enrichment Analysis (GSEA) were used for gene enrichment analyses and functional annotation.

### Analysis of relative gene expression and fungal biomass

After inoculation with *V. dahliae*, roots were collected at 0, 0.5, 1, 2, 4, 12, 24, 48, and 72 hours post-inoculation (hpi). The cDNA was obtained from the total RNA by reverse transcription, and a primer pair qGhHSFA4a-F/R was used to amplify *GhHSFA4a*. *UBQ* was used as the housekeeping gene (**Table S1**). Roots of *V. dahliae*-infected cotton were harvested at 0, 2, 12, and 72 hpi and the expression pattern of marker genes in four pathways was detected using qRT-PCR and primers for UBQ as well as the other marker genes (**Table S1**). Fungal biomass was quantified by extracting genomic DNA from *V. dahlia*e-infected roots at 15 days post-inoculation (dpi) for qRT-PCR using Vd-ITS-F/R (**Table S1**) for ribosomal RNA genes ITS1 and ITS2 (MT899267.1). The internal reference gene was *UBQ*. An ABI7500 Fast (Applied Biosystems, Waltham, MA, USA) instrument was used and the data were analyzed using the 2^-ΔΔCt^ method (Su et al., 2020).

### Genetic transformation of *A. thaliana*

The overexpression vector, pCAMBIA2300, was cleaved using BamHI and SalI restriction endonucleases. The CDS sequence of *GhHSFA4a*, amplified using 2300-GhHSFA4a-F/R (**Table S1**), was ligated to the cleaved pCAMBIA2300-35S-eGFP vector using In-Fusion Cloning and then inserted into *A. tumefaciens* GV3101. Transgenic *A. thaliana* was obtained using the floral dip method (Clough and Bent, 1998). Seeds were collected from the plants as well as sown, and the resultant seedlings were planted to screen for transgenic lines. DNA was extracted from the positive material and tested with eGFP-F/R, and the transcript levels were quantified using qGhHSFA4a-F/R and AtRubisco-F/R (**Table S1**). Relative gene expression was analyzed using the 2^-ΔΔCt^ method (Livak and Schmittgen, 2001).

### Statistical analyses

SPSS version 20.0 (IBM, Armonk, NY, USA) was used for all the statistical analyses. One-way analysis of variance (ANOVA) and *post hoc* Duncan’s multiple range test were used to analyze the data. Differences were considered statistically significant at *P* < 0.05.

## Results

### GhHSFA4a structure

**Figure 1:**
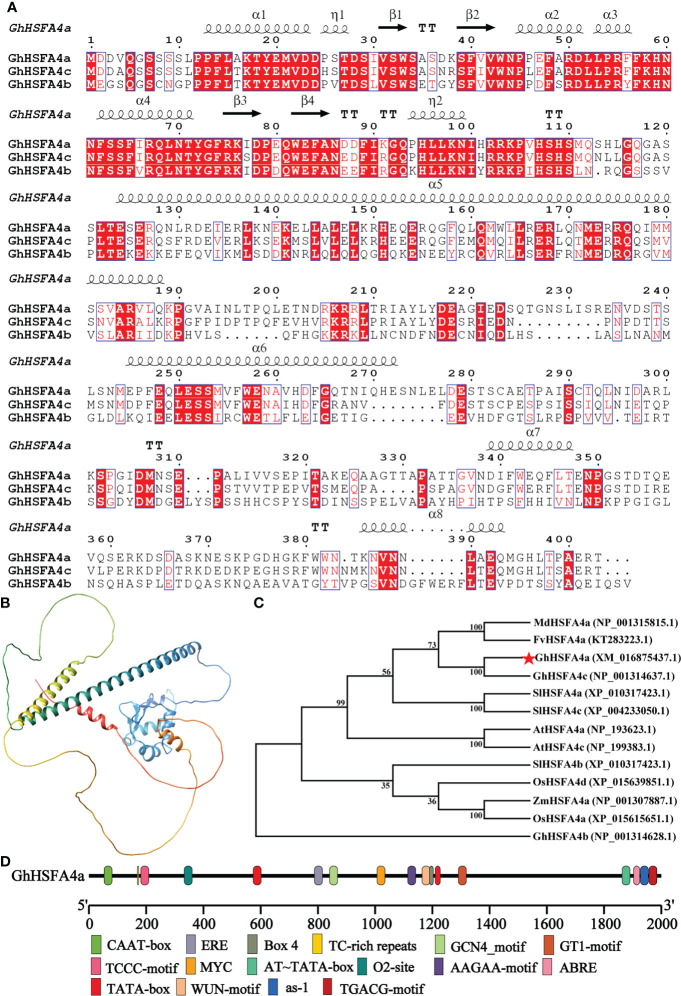
Structural characteristics of GhHSFA4a. **(A)** Alignment of GhHSFA4a, GhHSFA4b, GhHSFA4c proteins. **(B)** Three-dimensional GhHSFA4a model. **(C)** Phylogenetic tree of HSFA4 proteins from *G.hirsutum* (Gh), *Solanum lycopersicum* (SI), *A. thaliana* (At), *Fragaria vesca* (Fv), *Malus domestica* (Md), *Oryza sativa* (Os), and *Zea mays* (Zm). **(D)** Analysis of *cis*-acting element of the *GhHSFA4a* promoter region.

Based on our previous study, we cloned the 1212-bp full-length CDS of *GhHSFA4a*, which was identified using BlastP search IF. *GhHSFA4a*, which contains two exons, was mapped to chromosome D10 at *NC_053446.1*, and was shown to encode a protein of 403 amino acids with an estimated molecular mass of 46 kDa and an isoelectric point of 5.12. GhHSFA4a was found to contain an HSF-type DBD (**Figure S1**). Phylogenetic analysis of the fourth subcategory of HSFs A-type proteins in cotton and its homologous proteins from other plants showed that it was evolutionarily close to *F. vesca* HSFA4a (**Figure 1A**).

Multiple sequence alignments of the amino acid sequences of GhHSFA4a and other fourth subcategory A-type proteins were performed (**Figure 1B**). Its secondary structure is mainly composed of 37% alpha helix, 3% beta strand, and 58% disordered. The three-dimensional model showed that GhHSFA4a contains a solution NMR structure of heat shock factor protein 1 DBD from *Homo sapiens*, the Northeast Structural Genomics Consortium target HR3023C (**Figure 1C**).

A *cis*-element analysis of the sequence 2000- bp upstream of *GhHSFA4a* was performed to better understand its regulatory relationship at the transcriptional level (**Figure 1D**), which indicated that the GhHSFA4a promoter region contains hormone-related elements, such as the ABA response element (ABRE) and the methyl jasmonate (MeJA) response element (TGACG-motif), as well as other promoter *cis*-elements, such as the response defense and stress responsiveness element (TC-rich repeats), the light response element (TCCC-motif, Box 4), and elements associated with endosperm expression (GCN4_motif). Thus, *GhHSFA4a* might respond to biological stress through hormone induction.

### *GhHSFA4a* responds to *V. dahliae* stress

**Figure 2:**
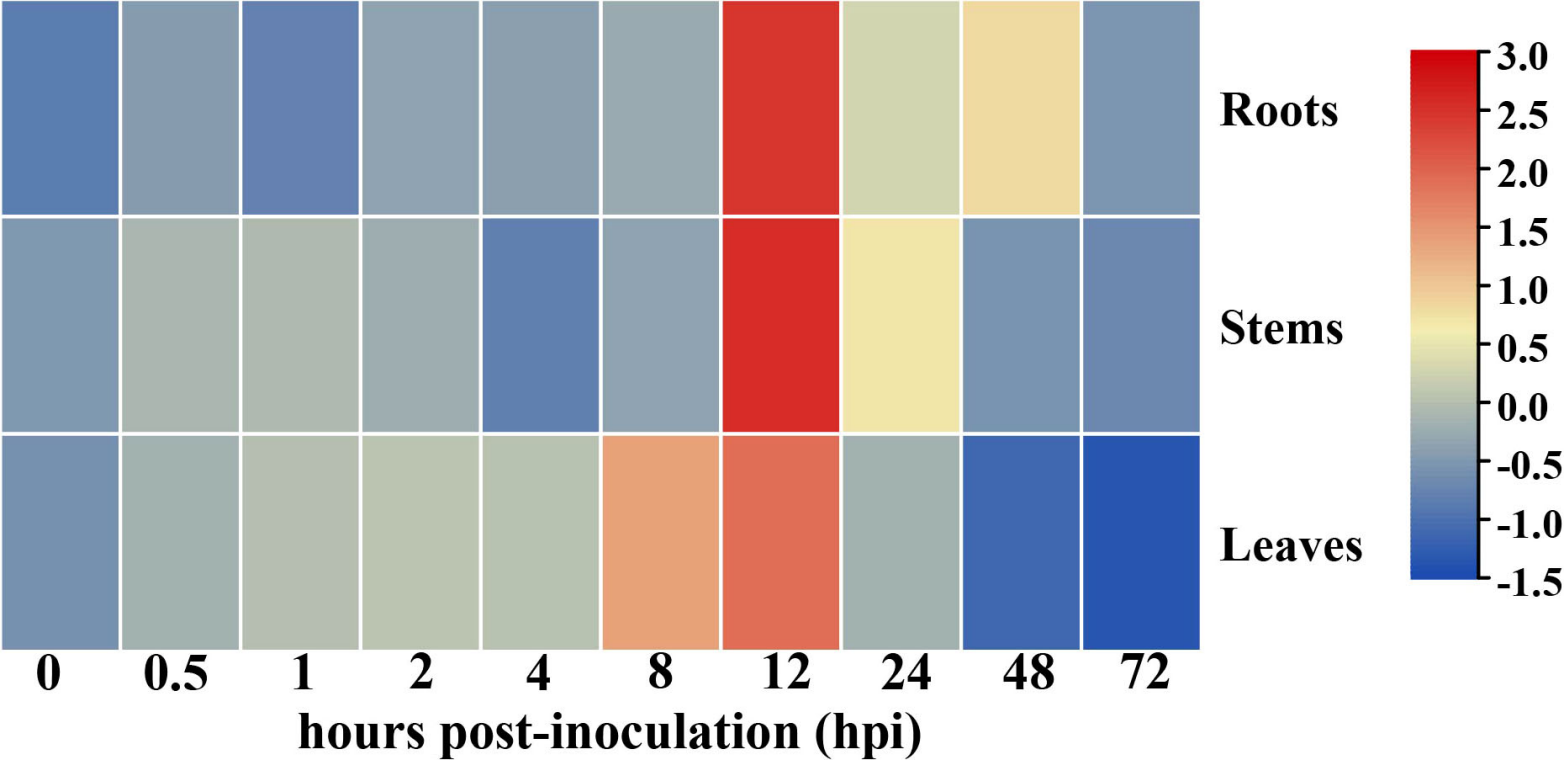
Expression of *GhHSFA4a* in cotton inoculated with *V. dahliae*. RNA was extracted from roots, stems, and leaves of 3-week-old seedings at 0, 0.5, 1, 2, 4, 8, 12, 24, 48, and 72 hours post-inoculation (hpi) with *V. dahliae*. Relative transcript levels were quantified using qRT-PCR with *UBQ* as the internal reference gene. The experiments were performed in duplicate using different RNA samples for the template.

The presence of *cis*-elements associated with immunity or defense reactions, including the ERF-box (Nishizawa-Yokoi et al., 2009), ABRE-box (Guo et al., 2016), and TGACG motif (Zander et al., 2012) within the promoter region of *GhHSFA4a* implies that the gene product may be involved in responses to pathogen infection. To test this assumption and investigate the role of *GhHSFA4a* in resistance to *V. dahliae* in cotton, we analyzed the expression of *GhHSFA4a* using qRT-PCR and discovered that expression was slightly induced at 0.5 hpi and peaked at 12 hpi. In a tissue-specific analysis, *GhHSFA4a* was constitutively expressed in cotton roots, stems, and leaves (**Figure 2**). These results indicate that *GhHSFA4a* can be induced after *V. dahliae* infection in cotton and might participate in cotton resistance to *V. dahliae.*

### GhHSFA4a is localized in the nucleus

**Figure 3:**
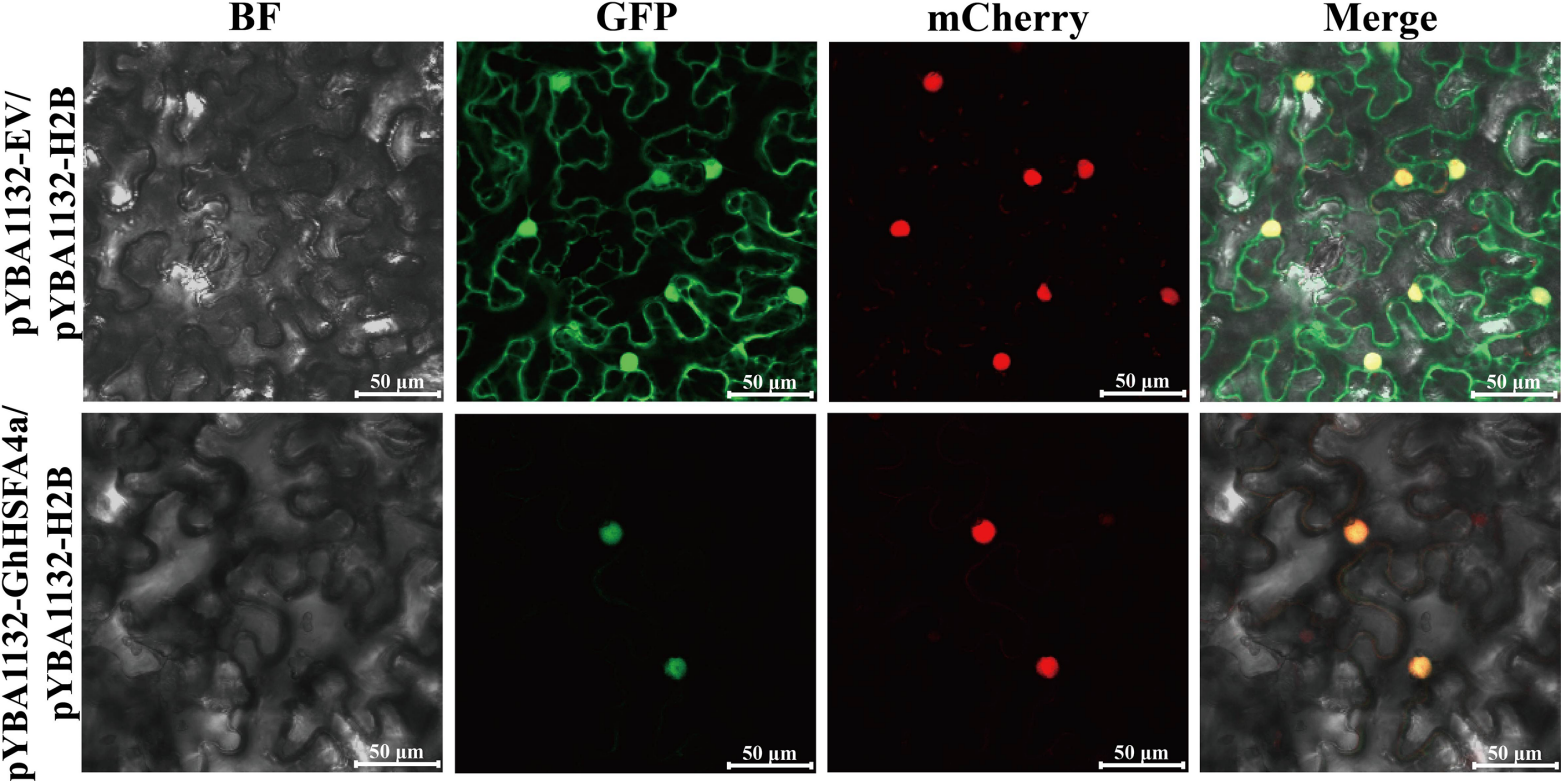
GhHSFA4a was localized in nucleus after transient overexpression of GhHSFA4a in leaves of *N. benthamiana*. *N. benthamiana* leaves were infiltrated with EHA105 cells containing pYBA1132::GhHSFA4a::GFP and using pYBA1132::GFP as a control. Shown are the confocal micrographs of *Agrobacterium*-infiltrated *N. benthamiana* leaves harvested at 48 hpi and counterstained with H2B-mCherry to localize GhHSFB4a-GFP. Bars = 50 μm.

To localize GhHSFA4a, we inserted a *pYBA1132*-controlled *GhHSFA4a-GFP* chimeric gene (*pYBA1132-GhHSFA4a-GFP*) or the control *pYBA1132-GFP* vector with H2B-mCherry as a nucleus marker into *N. benthamiana* leaves and then examined the cells for fluorescence using laser-scanning confocal microscopy at 48 hpi with *V. dahliae* (**Figure 3**). The GhHSFA4a-GFP fusion protein colocalized with H2B-mCherry exclusively within the nucleus. In contrast, in cells overexpressing pYBA1132-GFP, GFP signals were observed throughout the cell, including in the plasma membrane, cytoplasm, and nucleus. These results suggest that GhHSFA4a is localized to the nucleus and may be the TF responsible for activating the expression of downstream resistance genes.

### Silencing of *GhHSFA4a* enhanced sensitivity to *V. dahliae* in cotton

**Figure 4:**
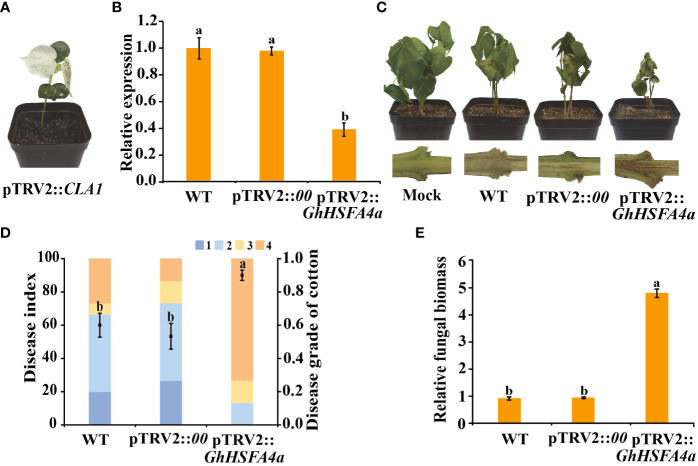
Silencing of *GhHSFA4a* attenuates plant resistance to *V. dahliae*. **(A)** Phenotype of pTRV2::*CLA1* at 14 days post *Agrobacterium* infiltration. **(B)** Expression of *GhHSFA4a* in pTRV2::*GhHSFA4a* and control plants. UBQ was the internal reference gene (2^-ΔΔCt^). **(C)** Disease phenotypes of *GhHSFA4a*-silenced plants at 15 dpi with *V. dahliae* strain V991. **(D)** Disease index of pTRV2::*GhHSFA4a* and control plants at 15 dpi with *V. dahliae*. **(E)** Fungal biomass determined using qRT-PCR at 15 dpi with *V. dahliae*. Error bars represent the standard deviations and different letters indicate significant differences at *p*<0.05.

To verify the function of *GhHSFA4a* in cotton resistance to *V. dahliae*, we used VIGS to reduce the expression of *GhHSFA4a* in Coker 312 cotton. One fully developed seedling cotyledon was infiltrated with pTRV2::*00* (negative control), and the other cotyledon was infiltrated with pTRV2*::GhHSFA4a*. After two weeks, strong photobleaching was present on newly emerged leaves on the seedlings transformed with pTRV2::*CLA1*, indicating that the VIGS system was successful under our experimental conditions (**Figure 4A**). *GhHSFA4a* expression levels were distinctly reduced in pTRV2::*GhHSFA4a* plants compared to those in pTRV2::*00* seedlings, and the qRT-PCR analysis indicated a silencing efficiency of 61% (**Figure 4B**). Two weeks after treatment with *V. dahliae*, the pTRV2::*GhHSFA4a* plants displayed more severe symptoms, such as wilted leaves and browned vascular bundles, than those observed in control plants (**Figure 4C**). The DI of *GhHSFA4a*-slienced cotton was significantly higher than that of the control cotton, and *GhHSFA4a* silencing was associated with the highest number of cotton plants with level 4 disease severity (**Figure 4D**). Supporting these results, the fungal biomass in silenced seedlings was 5-fold higher than that in control seedlings (**Figure 4E**). These results suggest that knockdown of *GhHSFA4a* attenuates the resistance of cotton plants to *V. dahliae.*

### RNA-seq revealed *GhHSFA4a* functions through GO, KEGG, and GSEA analysis

**Figure 5:**
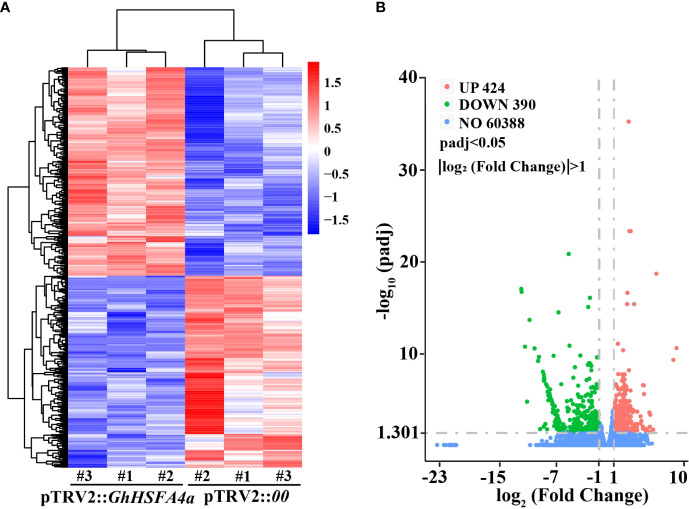
Analysis of DEGs between the transcriptomes of pTRV2::*00* and pTRV2::*GhHSFA4a* cottons. **(A)** Heat map of DEGs in each sample. **(B)** Volcano map of DEGs.

To investigate whether *GhHSFA4a* is involved in the disease resistance pathway, we used RNA-seq and analyzed the DEGs between pTRV2::*00* and pTRV2::*GhHSFA4a* seedlings (**Figure 5**). After filtering the original read quality, 38 Gb of clean sequence data were obtained for the two samples (5.81-6.72 Gb of clean reads for each sample). Using the *G. hirsutum* TM-1 transcriptome as the reference genome (ASM98774v1), 93.31-94.16% of the clean reads were mapped to the reference genome. Through standardization and screening of the read count, we identified 814 DEGs (424 upregulated and 390 down regulated). A heatmap was constructed for cluster analysis, which illustrates the global expression pattern of all DEGs and the repeatability between parallel groups (**Figure 5A**). DEGs were visualized using a volcano map, which showed the degree of difference among the 814 DEGs (**Figure 5B**). These DEGs reflect the response induced by *GhHSFA4a* knockdown.

**Figure 6:**
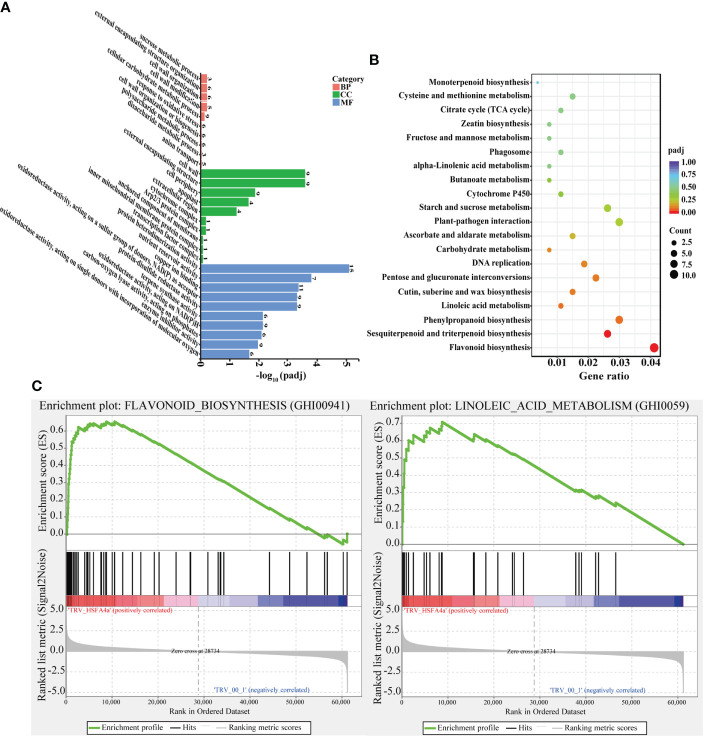
GO, KEGG, and GSEA metabolic pathway enrichment for DEGs in pTRV2::*00* and pTRV2::*GhHSFA4a* cotton. **(A)** GO terms. **(B)** KEGG terms. **(C)** GSEA analysis shows that two plant resistance-related gene sets are significantly activated in *GhHSFA4a*-silenced cotton compared with those in the control group. BP, biological process; CC, cellular component; MF, molecular function.

All DEGs between pTRV2::*00* and pTRV2::*GhHSFA4a* cotton were annotated for GO terms and classified into three functional categories. For biological process, “response to oxidative stress” revealed that DEGs were highly enriched in metabolic pathways closely related to disease resistance. For molecular function, “oxidoreductase activity”, “terpene synthase activity”, and “peroxidase activity” indicated that DEG were highly enriched for plant disease resistance pathways. Analysis of cellular components revealed that DEGs were associated with the “cell wall”, “external encapsulating structure”, “cell periphery”, and “apoplast” (**Figure 6A**). KEGG analysis showed that DEGs were enriched for 67 biological pathways in *G. hirsutum*. DEGs in pTRV2::*GhHSFA4a* cotton were enriched in pathways related to disease resistance, such as “flavonoid biosynthesis”, “sesquiterpenoid and triterpenoid biosynthesis”, “alpha-linolenic acid metabolism”, and “linoleic acid metabolism”. These functional annotations indicate that the disease resistance potential of cotton changed with *GhHSFA4a* silencing (**Figure 6B**). GSEA was performed using VIGS plants to further elucidate the function of *GhHSFA4a*. Compared to the gene sets in plants injected with the mock construct, 12 resistance-related gene sets were significantly activated in *GhHSFA4a*-silenced plants (e.g., “linoleic acid metabolism” and “flavonoid biosynthesis”; **Figure 6C**, **Table S2**). These results suggest that numerous genes in signaling and metabolic pathways involved in cotton disease resistance may be regulated by *GhHSFA4a* in a complex regulatory network for disease resistance. Downregulation of *GhHSFA4a* adversely affected related transcription and signaling pathways, seriously impairing disease resistance.

### Validation of some key genes in important pathways

**Figure 7:**
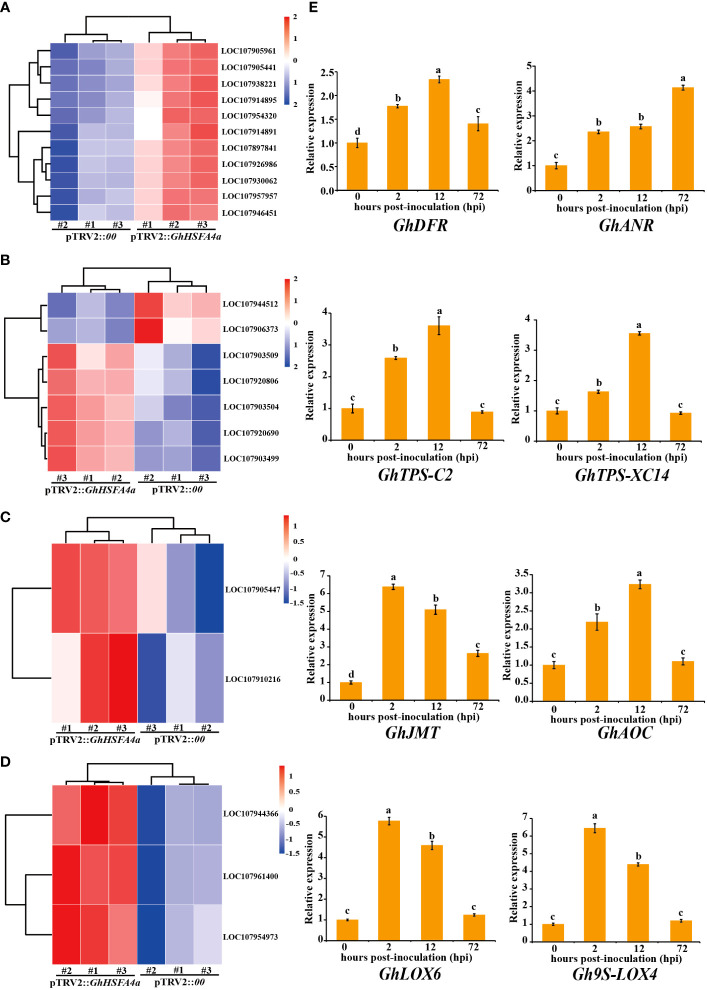
Validation of metabolic pathways significantly enriched for DEGs in pTRV2::*GhHSFA4a* cotton. Detailed expression profiles of genes related to **(A)** “flavonoid biosynthesis”, **(B)** “sesquiterpenoid and triterpenoid biosynthesis”, **(C)** “alpha-linolenic acid metabolism”, and **(D)** “linoleic acid metabolism”. **(E)** Relative expression levels over time of marker genes for the four pathways. Error bars represent the standard deviations and different letters indicate significant differences at *p*<0.05.

To analyze the relationship between metabolic pathways and resistance of cotton infected with *V. dahliae* using qRT-PCR, we selected marker genes dihydroflavonol 4-reductase (*GhDFR*, LOC107905441), anthocyanidin reductase (*GhANR*, LOC107905961), (+)-δ-cadinene synthase isozyme C2 (*GhTPS*-*C2*, LOC107920690), (+)-δ-cadinene synthase isozyme XC14 (*GhTPS*-*XC14*, LOC107920806), jasmonic acid carboxyl methyltransferase (*GhJMT*, LOC107905447), allene oxide cyclase (*GhAOC*, LOC107910216), lipoxygenase 6 (*GhLOX6*, LOC107961400), and linoleate 9S-lipoxygenase 4 (*Gh9S-LOX4*, LOC107954973), that responded to “flavonoid biosynthesis”, “sesquiterpenoid and triterpenoid biosynthesis”, “linoleic acid metabolism”, and “alpha-linolenic acid metabolism” pathways. The expression of these eight genes was consistently upregulated in *V. dahliae-*infected plants; however, peak expression occurred at different times (**Figure 7**). These results indicate that *GhHSFA4a* contributed to cotton resistance to *V. dahliae* by modulating the four DEG-enriched pathways.

### Overexpression of *GhHSFA4a* enhanced *V. dahliae* resistance in *Arabidopsis*

**Figure 8:**
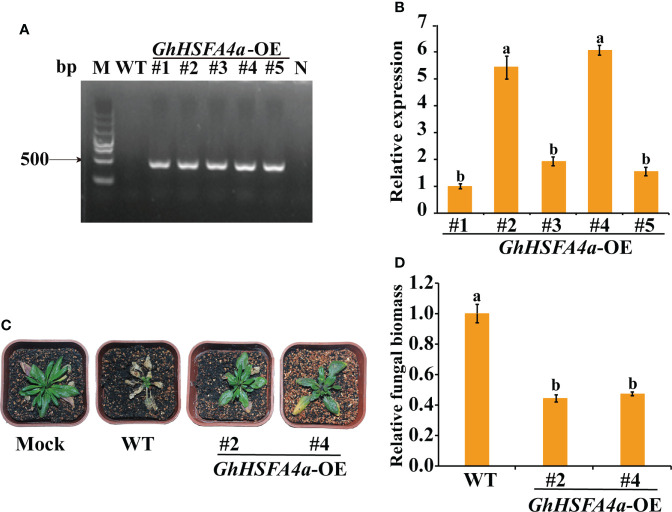
Enhanced *V. dahliae* resistance of *Arabidopsis* plants overexpressing *GhHSFA4a*. **(A)** PCR amplicons of *GhHSFA4a* in positive transgenic *Arabidopsis*. **(B)** Expression levels of *GhHSFA4a* in transgenic *Arabidopsis* lines (#1-#5). *AtRubisco* was used as an internal control. **(C)** Symptoms in wild-type and two *GhHSFA4a*-overexpressing (OE) lines of *Arabidopsis* plants at 21 dpi with *V. dahliae.* **(D)** Fungal biomass determined using qRT-PCR in wild-type (WT) and transgenic *Arabidopsis* plants. Error bars represent the standard deviations and different letters indicate significant differences at *p*<0.05.

To further assess the function of *GhHSFA4a* in *V. dahliae* resistance, *Arabidopsis* seedlings were transformed with *GhHSFA4a* using *Agrobacterium* harvard recombinant plasmid with the CDS of *GhHSFA4a* and the 35S promoter. The expression levels of *GhHSFA4a* in five positive *GhHSFA4a*-transgenic lines were determined using PCR and qRT-PCR (**Figures 8A, B**). T3 homozygous generations of two independent *GhHSFA4a*-transgenic lines (OE-2 and OE-4) with high expression levels of *GhHSFA4a* were further analyzed. Wild-type and transgenic *Arabidopsis* plants were inoculated with the *V. dahliae* strain V991 and symptoms were monitored over time until 21 dpi. The transgenic plants had less wilting, chlorosis, and fungal biomass (**Figures 8C, D**); thus, supporting the view that GhHSFA4a positively contributes to plant resistance to *V. dahliae.*

## Discussion

Plant HSFs are integral to the complex regulatory systems that coordinate plant development and molecular responses to biotic and abiotic stresses (Li et al., 2014). A few members of class A HSFs mainly play a positive regulatory role in plant abiotic stress such as heat (Liu et al., 2011; Shi et al., 2015; Meena et al., 2022; Zheng et al., 2022). HSFA4 can enhance plant tolerance to heat, high salinity, cadmium, light, and oxidative stress in *Arabidopsis*, rice, wheat, and other plants (Shim et al., 2009; Lang et al., 2017; Chen et al., 2018; Huang et al., 2018; Li et al., 2018; Andrási et al., 2019). We previously identified a set of DEGs in the transcriptome and metabolomic profiles of *Arabidopsis* plants inoculated with *V. dahliae* (Su et al., 2018). One of these DEGs is homologous to *G. hirsutum GhHSFA4a*, which encodes a TF belonging to the HSF family. Therefore, we hypothesized that *GhHSFA4a* might be involved in the resistance of cotton to *V. dahliae* and here, we characterized its biological functions.

Structural characteristics revealed that HSFA class proteins usually contain DBD, HR-A/B region, NLS as well as NES motifs, and AHA domains (Scharf et al., 1998; Scharf et al., 2012). GhHSFA4a contains a conserved DBD domain (**Figure S1**). We visualized the *GhHSFA4a*-GFP fusion protein in the nucleus, a feature consistent with the location of HSFs identified in previous studies (**Figure 3**) (Gai et al., 2020; Zhang et al., 2020). Our phylogenetic analysis indicated that GhHSFA4a might have a function similar to that of *F. vesca* HSFA4a (**Figure 1C**). Additionally, the *cis*-element analysis showed that the *GhHSFA4a* promoter region consists of several elements that respond to defense, stress, and hormones (**Figure 1D**). Based on our bioinformatic analysis and previous research related to HSFA4a, we hypothesized that GhHSFA4 may confer resistance to *V. dahliae* in cotton by modulating a defense pathway.

Plants protect themselves against microbes through structural resistance and a sophisticated immune system that activates defense responses. Numerous lines of evidence support the hypothesis that HSFs are required for plant pathogen resistance (Kumar et al., 2009; Daurelio et al., 2013). However, few studies have been conductedon HSFs and their involvement in cotton resistance against *V. dahliae*. In our study, *GhHSFA4a* expression was significantly induced at 12 hpi, an early stage of *V. dahliae* infection (**Figure 2**). The HSF-like transcription factor *TB1* acts as a major molecular switch from plant growth to defense by binding to the *TL1* (GAAGAAGAA) *cis*-element required for the induction of endoplasmic-reticulum-resident genes (Pajerowska-Mukhtar et al., 2012). Previous studies have shown that microbes such as *Pseudomonas syringae* and various fungal pathogens activate a well-defined subset of HSF genes, including *HSFA2*, *HSFA4A*, *HSFA8*, *HSFB1*, and *HSFB2B* (Andrási et al., 2021). In addition, *HSFA4A/HSFA5* factors have been implicated in cell death triggered by pathogenic infection (Baniwal et al., 2007).

In rice, the *spl7* mutation disrupts the *HSFA4* gene, leading to enhanced heat- and UV- light-induced cell necrosis and susceptibility to several pathogens (Yamanouchi et al., 2002). Overexpression of the transcription factor MYB49 in tomatoes decreases the DI and enhances resistance to *Phytophthora infestans* (Cui et al., 2018). The VIGS-mediated silencing of cyclic nucleotide-gated ion channels (CNGCs) *MdCNGC2* in fruits improves resistance to *Botryosphaeria dothidea* (Zhou et al., 2020). These results are consistent with our findings from the VIGS of *GhHSFA4a* in cotton and the overexpression of *GhHSFA4a* in *Arabidopsis*. The increased DI and fungal biomass in cotton after silencing *GhHSFA4a* in seedlings was evidence that resistance was compromised (**Figure 4**). Alternatively, overexpression of *GhHSFA4a* in *Arabidopsis* enhanced its resistance to *V. dahliae* (**Figure 8**). Our preliminary results support the prediction that *GhHSFA4a* participates in *V. dahliae* resistance. However, the regulatory network involved in *GhHSFA4a* in cotton disease resistance needs to be elucidated.

A complex regulatory network of flavor-related metabolites was revealed in *Actinidia chinensis* through the analysis of metabolome and transcriptome data, which helped identify key structural genes and TFs that regulate the metabolism of soluble sugars, organic acids, and important volatiles (Wang et al., 2022). In non-stressed *HSFA1b*-overexpressing (*HSFA1bOx*) *Arabidopsis*, the expression of 509 genes involved in responses to biotic stress were altered (Bechtold et al., 2013). Following our RNA-seq analysis of the transcriptome of *GhHSFA4a*-silenced plants meant to identify the molecular role of GhHSFA4a after inoculation with *V. dahliae*, we identified 814 DEGs between pTRV2::*00* and pTRV2::*GhHSFA4a* plants; 424 genes were upregulated and 390 genes were downregulated after *GhHSFA4a*-silencing (**Figure 5**).

The KEGG analysis revealed that DEGs were mainly enriched in pathways for the synthesis of secondary metabolites (“flavonoid biosynthesis”, “sesquiterpenoid and triterpenoid biosynthesis”; **Figure 6B**). Flavonoids are involved in plant development as well as defense and help plants adapt to environmental conditions (Hichri et al., 2011). Triterpenoids are considered to be defensive compounds against pathogens (Vincken et al., 2007; Szakiel et al., 2011). *Nbnrp1* mediates PevD1-induced defense responses by regulating the sesquiterpenoid phytoalexin biosynthesis pathway (Liang et al., 2021). In our study, DEGs were involved in plant hormone-related pathways, such as “linoleic acid metabolism” and “alpha-linolenic acid metabolism” (**Figure 6B**). Linolenic acid and linoleic acid are key components in the synthesis of the hormones jasmonic acid (JA) and MeJA, which have numerous physiological functions in disease resistance such as direct inhibition of pathogens, transmission of resistance signals, and induction of defense compound synthesis (Creelman et al., 1992; Gundlach et al., 1992; Harms et al., 1995; Singh et al., 2011). *Arabidopsis* mutants *fad3*-2, *fad7*-2, and *fad8*, which lack linolenic acid, express low JA contents, and the expression of JA-related genes is not induced. These mutants are sensitive to *Pythium mastophorum*, and their antifungal ability is restored after MeJA application (Vijayan et al., 1998).

Based on our transcriptomic results, we hypothesize that GhHSFA4a is involved in cotton disease resistance by regulating the synthesis of secondary metabolites and plant hormones. This hypothesis is supported by the qRT-PCR results that showed that eight marker genes for “flavonoid biosynthesis”, “sesquiterpenoid and triterpenoid biosynthesis”, and “alpha-linolenic acid metabolism” pathways were induced by *V. dahliae* (**Figure 7**). In previous studies, these marker genes were identified as key genes in the above pathways and were involved in stress resistance. For example, in tobacco, the overexpression of *DFR* or *ANR*, which encode crucial regulatory enzymes for flavonoid synthesis, improves the resistance of plants to biotic or abiotic stresses (Tan et al., 2018; Sun et al., 2021). The transcription levels of TPS family genes and jasmonate-responsive genes in cotton plants treated with plant-growth-promoting rhizobacteria were higher than those in the untreated control plants and exhibited improved resistance to *Spodoptera exigua* (Zebelo et al., 2016). In *Arabidopsis*, JMT catalyzes the conversion of JA into MeJA and participates in JA-responsive defense mechanisms (Seo et al., 2001). In *Arachis hypogaea*, *AhAOC* can be induced by JA to enhance plant resistance to stress (Liu et al., 2015). Tobacco *NaLOX3* expression is induced by the fungal initiator ergosterol (Dadakova et al., 2013), while *Pisum sativum* L enhances waterlogging tolerance by regulating linoleate *Ps9S-LOX5* gene expression (Zaman et al., 2019).

## Conclusions

In this study, *GhHSFA4a*, encoding a heat shock factor located in the nucleus, was induced by *V. dahliae* and was constitutively expressed in the roots, stems, and leaves of cotton. When *GhHSFA4a* was silenced, cotton resistance to *V. dahliae* was compromised, whereas its overexpression in *Arabidopsis* significantly improved resistance. Furthermore, *GhHSFA4a* was shown to be involved in “flavonoid biosynthesis”, “sesquiterpenoid and triterpenoid biosynthesis”, “linoleic acid metabolism” and “alpha-linolenic acid metabolism” pathways. In summary, GhHSFA4a contributes positively to plant resistance against *V. dahliae* and mediates flavonoid and terpenoid secondary metabolic pathways and JA biosynthesis and signaling pathways, indicating that it is a potential candidate gene for molecular genetic breeding.

## Data availability statement

The original contributions presented in the study are publicly available. This data can be found here: NCBI, PRJNA877353.

## Author contributions

Conceptualization, XS and EL. Methodology, HL. Software, HL. Validation, HC. Formal analysis, LL and DW. Investigation, LL and DW. Resources, HG. Data curation, LL and DW. Writing—original draft preparation, LL and DW. Writing—review and editing, XS and LL. Visualization, EL. Supervision, EL. Funding acquisition, XS, HC, and HL All authors contributed to the article and approved the submitted version.

## Funding

This research was supported by Hainan Yazhou Bay Seed Laboratory (project of B21HJ0215), the National Natural Science Foundation of China (32072376 and 32160624), and Central Public-interest Scientific Institution Basal Research Fund (No. Y2022CG05).

## Conflict of interest

The authors declare that the research was conducted in the absence of any commercial or financial relationships that could be construed as a potential conflict of interest.

## Publisher’s note

All claims expressed in this article are solely those of the authors and do not necessarily represent those of their affiliated organizations, or those of the publisher, the editors and the reviewers. Any product that may be evaluated in this article, or claim that may be made by its manufacturer, is not guaranteed or endorsed by the publisher.
