# Supplementary material for: The heat shock factor GhHSFA4a positively regulates cotton resistance to Verticillium dahliae
Source: Front Plant Sci. 2022 Nov 3;13:1050216. doi: 10.3389/fpls.2022.1050216 (PMC9669655; doi:10.3389/fpls.2022.1050216)
Supplement: Supplementary file 1 [file DataSheet_1.docx]

Supplementary Material

Table S1. The primers used in this study^1^

| **Construct name** | **Primer name** | **Primer sequence (5＇-3＇)** |
| --- | --- | --- |
| pYBA1132 | 1132-GhHSFA4a-F | TCTAGAACTAGT**GGATCC**ATGGATGATGTTCAGGGCAG |
|  | 1132-GhHSFA4a-R | TAAGCTTGATATC**GAATTC**CGTTCTCTCCGCAGGAGTAAG |
| pTRV2 | pTRV2-GhHSFA4a-F | TCTGTGAGTAAGGTTACC**GAATTC**CAGGAGCGTCAAGGATTTCAGTT |
|  | pTRV2-GhHSFA4a-R | ACGCGTGAGCTCGGTACC**GGATCC**CCCAGTTGTTGCAGGTGCAGTAG |
| pCAMBIA2300-35S-OCS | 2300-GhHSFA4a-F | GGACAGGGTACCCGG**GGATCC**ATGGATGATGTTCAGGGCAGC |
|  | 2300-GhHSFA4a-R | CACCATGGTACTAGT**GTCGAC**CGTTCTCTCCGCAGGAGTAAGA |
| RT-qPCR | qGhHSFA4a-F | TTGCCATAAATCTGACACCAC |
|  | qGhHSFA4a-R | TCATGTACCGCATTCTCCC |
|  | UBQ-F | AGCTCGGATACGATTGATAACG |
|  | UBQ-R | GAAGACGAAGAACAAGGGGAAG |
|  | Vd-ITS-F | TCCGTAGGTGAACCTGCGG |
|  | Vd-ITS-R | TCCTCCGCTTATTGATATGC |
|  | AtRubisco-F | GCAAGTGTTGGGTTCAAAGCTGGTG |
|  | AtRubisco-R | CCAGGTTGAGGAGTTACTCGGAATGCTG |
|  | qGhDFR-F | GCGACCCTGACAACTCGAAGAA |
|  | qGhDFR-R | GACTCGAAGTCCATAGGCGTGG |
|  | qGhANR-F | TGATCAAACCAGCGACCCAAGG |
|  | qGhANR-R | CCAAGCTGCCTTTTCAGCCAAC |
|  | qGhTPS-C2-F | TGCCGAGAACGACCTCTACACT |
|  | qGhTPS-C2-R | CCTCGAACATCGCTTATCACGGA |
|  | qGhTPS-XC14-F | ACTTTGAGCCCCAATATTCTCTTGGT |
|  | qGhTPS-XC14-R | TTGTCTCCCATGCTTAGCCACC |
|  | qGhJMT-F | GGACATAGTACGAGCGACGAGC |
|  | qGhJMT-R | GGAGGGTGCCAATCCAATACCC |
|  | qGhAOC-F | ATACGAAGGGCTCCGGATCGAA |
|  | qGhAOC-R | CATCTCCCCTGCTTTCACCTGG |
|  | qGhLOX6-F | AGGTCAGTGTCAGATCCAAGTGA |
|  | qGhLOX6-R | GCAATTGAGCACTGACATGTTGT |
|  | qGh9S-LOX4-F | GTCGGAAATTCGAAACGTCGGC |
|  | qGh9S-LOX4-R | CGGTTTGGAGGATAACCTGCGT |
| PCR | eGFP-F | CCTGAAGTTCATCTGCACCAC |
|  | eGFP-R | GACTGGGTGCTCAGGTAGTG |

1. The restriction recognition sequences of BamHI (GGATCC), EcoRⅠ(GAATTC) and SalⅠ (GTCGAC) within the primers are in bold and underlined. Genes included in qPCR assay are *GhHSFA4a* (XM_016875437.1) *UBQ* (LOC107925174), *Vd-ITS* (MT899267.1), *AtRubisco* (ATCG00490), *GhDFR* (LOC107905441), *GhANR* (LOC107905961), *GhTPS-C2* (LOC107920690), *GhTPS-XC14* (LOC107920806), *GhJMT* (LOC107905447), *GhAOC* (LOC107910216), *GhLOX6* (LOC107961400), *Gh9S-LOX4* (LOC107954973).

Table S2 The significantly enriched pathway of GSEA analysis in GhHSFA4a-slienced cotton

| ID | NES | NOM p-val |
| --- | --- | --- |
| LINOLEIC_ACID_METABOLISM (GHI00591) | 1.769 | 0 |
| FLAVONOID_BIOSYNTHESIS (GHI00941) | 1.662 | 0 |
| PHENYLALANINE_TYROSINE_AND_TRYPTOPHAN_BIOSYNTHESIS (GHI00400) | 1.661 | 0 |
| GLYCOSYLTRANSFERASES (GHI01003) | 1.658 | 0 |
| PYRUVATE_METABOLISM (GHI00620) | 1.639 | 0 |
| STRUCTURAL_PROTEINS (GHI99992) | 1.628 | 0 |
| EXOSOME (GHI04147) | 1.538 | 0 |
| AMINO_SUGAR_AND_NUCLEOTIDE_SUGAR_METABOLISM (GHI00520) | 1.471 | 0 |
| ASCORBATE_AND_ALDARATE_METABOLISM (GHI00053) | 1.390 | 0 |
| NITROGEN_METABOLISM (GHI00910) | 1.366 | 0 |
| PENTOSE_AND_GLUCURONATE_INTERCONVERSIONS (GHI00040) | 1.323 | 0 |
| ENZYMES_WITH_EC_NUMBERS (GHI99980) | 1.272 | 0 |


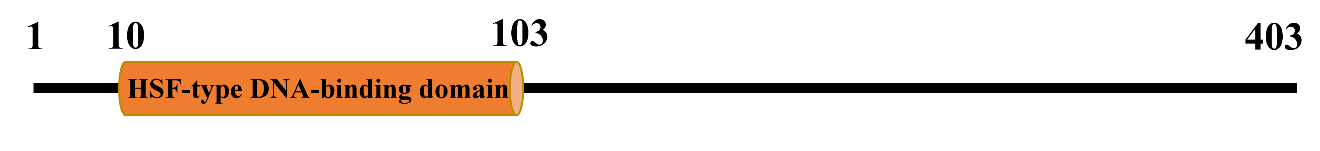


Figure S1 GhHSFA4a conserved domain^2^

1. Conserved domain is highlighted in orange.
